# Small population size and extremely low levels of genetic diversity in island populations of the platypus, *Ornithorhynchus anatinus*

**DOI:** 10.1002/ece3.195

**Published:** 2012-04

**Authors:** Elise Furlan, J Stoklosa, J Griffiths, N Gust, R Ellis, R M Huggins, A R Weeks

**Affiliations:** 1 Department of Genetics, The University of MelbourneParkville, Victoria 3010, Australia; 2 Department of Mathematics and Statistics, The University of MelbourneParkville, Victoria 3010, Australia; 3 CESAR293 Royal Parade, Parkville, Victoria 3052, Australia; 4 Department of Primary Industries, Parks, Water and Environment, Resource Management and Conservation DivisionTasmania 7001, Australia; 5 Department of Environment and Natural ResourcesSouth Australia 5001, Australia

**Keywords:** Conservation, genetic diversity, island populations, platypus, *Ornithorhynchus anatinus*

## Abstract

Genetic diversity generally underpins population resilience and persistence. Reductions in population size and absence of gene flow can lead to reductions in genetic diversity, reproductive fitness, and a limited ability to adapt to environmental change increasing the risk of extinction. Island populations are typically small and isolated, and as a result, inbreeding and reduced genetic diversity elevate their extinction risk. Two island populations of the platypus, *Ornithorhynchus anatinus*, exist; a naturally occurring population on King Island in Bass Strait and a recently introduced population on Kangaroo Island off the coast of South Australia. Here we assessed the genetic diversity within these two island populations and contrasted these patterns with genetic diversity estimates in areas from which the populations are likely to have been founded. On Kangaroo Island, we also modeled live capture data to determine estimates of population size. Levels of genetic diversity in King Island platypuses are perilously low, with eight of 13 microsatellite loci fixed, likely reflecting their small population size and prolonged isolation. Estimates of heterozygosity detected by microsatellites (*H*_E_= 0.032) are among the lowest level of genetic diversity recorded by this method in a naturally outbreeding vertebrate population. In contrast, estimates of genetic diversity on Kangaroo Island are somewhat higher. However, estimates of small population size and the limited founders combined with genetic isolation are likely to lead to further losses of genetic diversity through time for the Kangaroo Island platypus population. Implications for the future of these and similarly isolated or genetically depauperate populations are discussed.

## Introduction

Genetic diversity has been identified as an important factor influencing a population's long-term potential for survival (Bouzat 2010). The contribution of genetic diversity has been recognized in numerous aspects of population persistence, and is critical for long-term fitness and adaptation [see Frankham (2005) for a review]. A loss of genetic diversity has been shown to affect individual fitness with decreased sperm quality (Hedrick and Fredrickson 2010), reduced litter size (Hedrick and Fredrickson 2010), increased juvenile mortality (Ralls et al. 1988), and increased susceptibility to disease and parasites (Coltman et al. 1999). Accordingly, populations lacking genetic diversity often exhibit an increased rate of extinction (Markert et al. 2010). Inbreeding, genetic drift, restricted gene flow, and small population size all contribute to a reduction in genetic diversity. Fragmented and threatened populations are typically exposed to these conditions, which is likely to increase their risk of extinction (Saccheri et al. 1998; Madsen et al. 1999; Frankham et al. 2010).

Island populations are often isolated and small in size, and therefore experience increased levels of inbreeding and a greater impact of genetic drift. As a result, these populations generally have lower levels of genetic diversity and fitness than counterparts found in mainland populations. For example, the black-footed rock-wallabies of Barrow Island, Western Australia, have extremely low levels of genetic diversity (*H*_E_= 0.053), which has led to this population suffering inbreeding depression: females experience reduced fecundity and individuals exhibit increased levels of fluctuating asymmetry (Eldridge et al. 1999). These findings are not atypical with island populations frequently identified as having reduced genetic diversity, increased levels of inbreeding, and therefore a higher extinction risk (Frankham 1997).

**Figure 1:**
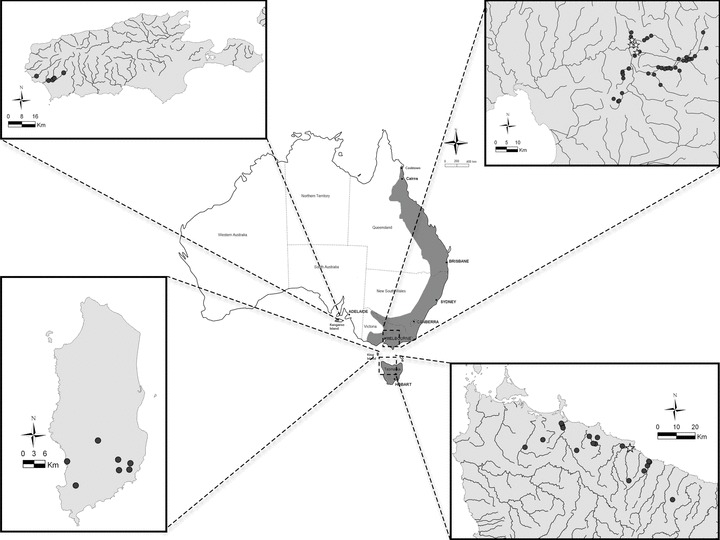
Gray shading indicates the current distribution of *Ornithorhynchus anatinus* throughout Australia. Inserts show, clockwise from top right; upper Yarra, Victoria; northwestern Tasmania; King Island, Tasmania; and Kangaroo Island, South Australia. Sampling locations are indicated by black dots. White stars indicate the approximate location of Kangaroo Island founders from Tasmania and Victoria.

The platypus, *Ornithorhynchus anatinus*, is found in eastern Australia, from as far north as Cooktown in Queensland, along the east coast to Victoria and throughout Tasmania (Fig. 1). Two island populations of *O. anatinus* exist within this distribution; King Island in Bass Strait, and Kangaroo Island off the coast of South Australia. The population on King Island is naturally occurring and likely dates back to the existence of a land bridge across Bass Strait connecting Tasmania with the mainland. Intermittent rises and falls in sea levels have seen this region periodically disconnect and reconnect with the mainland until its final isolation about 11,800 years ago (Blom 1988). King Island remained connected to Tasmania until approximately 10,000 years ago (Blom 1988). Despite its more recent connection with Tasmania, preliminary microsatellite analysis indicate platypuses to be more closely related to Victorian individuals (Akiyama 1998).

The Kangaroo Island platypus population was established with introductions from both Tasmanian and Victorian individuals. In 1928, one female and two males were transferred from Wynyard, Tasmania (Anonymous 1934; Flora and Fauna Board of South Australia Report, 1928–1929). This was followed by several introductions from Healesville in Victoria with five males and five females relocated in 1941 (Anonymous 1941; Fleay 1941; Anonymous 1946) and three males and three females in 1946 (Anonymous 1946). Today, Kangaroo Island is home to a self-sustaining population of platypuses. Akiyama (1998) conducted a preliminary genetic investigation of Kangaroo Island platypuses using microsatellite markers. Genetic diversity appeared to be relatively high, but the absence of comparable analyses for other platypus populations makes the interpretation of these results difficult. The contribution of founding individuals from both Tasmania and Victoria remains contentious. Previous studies have revealed mitochondrial DNA (mtDNA) and microsatellite markers to be more similar to Victorian individuals (Gemmell 1994; Akiyama 1998) but, critically, comparisons were made to individuals outside the regions from which founders were sourced. Current levels of genetic diversity in the island population and the persistence of Tasmanian and Victorian genes have yet to be adequately addressed.

Kangaroo Island imposes a restricted area of suitable aquatic habitat and isolation from other platypus populations. As a consequence, small population size and a lack of naturally occurring gene flow are likely to lead to inbreeding, the fixation of alleles, and associated reductions in genetic diversity over time. In addition, the limited founder number is likely to contribute to problems associated with inbreeding. Distribution records on Kangaroo Island suggest that platypuses are confined to the western end of the island (Fig. 1), further restricting population growth. Knowledge of the population size on the island can further inform estimates of genetic diversity and give some indication of population fitness. Population size relates to genetic variation (Frankham 1996) and is also significantly correlated with fitness (Reed and Frankham 2003). Consequently, small population size can reduce the evolutionary potential of a species (Frankham 1996). Additionally, effective population size can be inferred from population size estimates (Caballero 1994) and be used to predict the loss of genetic variation over time (Frankham 1995b). Due to the limitation of suitable habitat on Kangaroo Island, population size is likely to be small and associated threats to genetic variation and population fitness are anticipated.

Here we aim to investigate current levels of genetic diversity on King Island and Kangaroo Island by genotyping individuals at 13 polymorphic microsatellite markers described in Furlan et al. (2010). We also sequence fragments of the mitochondrial genes *cytochrome oxidase* subunit II and *cytochrome b* to determine the relationship between King Island and Tasmania or Victoria and the contributions of founding individuals on Kangaroo Island. In addition, live capture data are modeled to determine population size estimates for Kangaroo Island and provide insight into the likely future losses of genetic diversity in this population.

## Methods

### Genetic analysis

Platypus live-capture surveys were conducted using either gill nets [refer to Grant and Carrick (1974) for methodologies] or fyke nets. Fyke nets were set in pairs, back to back with the cod end secured well above water level. Nets were set approximately 2 h before sunset and checked regularly throughout the night. Individuals were identified by a uniquely coded passive integrated transponder tag (Trovan, Trovan Ltd., East Yorkshire, UK, or Allflex, Allflex Australia, Brisbane, Australia). Trapped individuals were scanned for a tag and any untagged individuals had one inserted subcutaneously between the scapulae following protocols in Grant and Whittington (1991). Each unique individual had a section of skin webbing approximately 2 mm^2^ cut from the distal margin of webbing on the rear foot using sharp, sterile scissors. All samples were immediately placed in 2 mL Eppendorf (Hamburg, Germany) tubes containing 100% ethanol.

Platypus web-tissue samples were collected from 12 individuals on Kangaroo Island between December 2008 and December 2009. Eleven platypuses were sourced from Rocky River and one from Breakneck River (Fig. 1), representing the only two rivers in which platypus presence has been confirmed on the Island. These reside within the Flinders Chase National Park and the adjoining Ravine des Casoars Wilderness Protection Area, supporting a total land area of 739 km^2^ (Smith 1995). Additional samples were collected from Kangaroo Island (*n*= 3) and Warrawong Sanctuary (*n*= 10) between 1991 and 1996 as part of a previous study (Akiyama 1998). Warrawong Sanctuary is located on mainland South Australia and represents a captive population sourced from Kangaroo Island individuals. Three of the sampled “Warrawong Sanctuary” individuals were originally sourced from Kangaroo Island, while the remaining seven individuals were subsequent generations bred within the sanctuary. Eighteen individuals were sampled from King Island in January 2009 (Fig. 1). Additional deceased samples (*n*= 3) were obtained opportunistically between 2000 and 2007. The island encompasses around 1098 km^2^ of land, although platypuses are primarily restricted to the rivers flowing to the east and south (Barnes et al. 2003). On the mainland, 60 individuals were sourced from the upper Yarra catchment in Victoria (hereafter referred to as *Victoria*) between 2008 and 2010 (Fig. 1). In Tasmania, 19 individuals were sampled from Duck, Black-Detention, Inglis, Cam, and Leven catchments in the states northwest (hereafter referred to as *Tasmania*) between 2007 and 2009 (Fig. 1). Tasmanian and Victorian individuals have been sampled from areas that potentially provided founding individuals for Kangaroo Island and have been included here for comparative purposes.

DNA was extracted from web-tissue using a cetyl trimethylammonium bromide (CTAB)-phenol/chloroform extraction (Sambrook and Russell 2001) and genotyped at 13 microsatellite loci (Furlan et al. 2010) and two partial mitochondrial genes. Reactions contained 2 µl of template DNA, 1× polymerase reaction buffer, 2 mM MgCl_2_, 0.2 mM deoxyribonucleotide triphosphates (dNTPs), 0.5 mg/mL bovine serum albumin (BSA) (New England Biolabs, Ipswich, MA), 0.03 U of *Taq* DNA polymerase (New England Biolabs), 0.3 µM forward primer end-labeled with [c33P] adenosine triphosphate (ATP), 0.2 µM unlabeled forward primer, and 0.5 µM unlabeled reverse primer. Reactions were made up to a final volume of 10 µl with ddH_2_O. Polymerase chain reactions (PCRs) were carried out with an Eppendorf Mastercycler (Eppendorf, Hamburg, Germany). Samples underwent initial denaturation at 95°C for 10 min, followed by 35 cycles of 95°C for 20 sec, 55°C for 30 sec, and 72°C for 30 sec. A final extension step of 72°C for 3 min completed the reaction. PCR products were then run through 5% polyacrylamide denaturing gels at 65 W for 2–4 h and exposed to autoradiography film (OGX, CEA, Sweden) for one to five days. Alleles were sized by comparison with λgt11 ladders (Promega fmol_ DNA Cycle Sequencing System, Madison, WI).

Mitochondrial DNA primers were derived from the published *O. anatinus* mitochondrial genome (Warren et al. 2008). A subset of samples was analyzed from each location. Sequences were amplified to obtain a 603 bp segment of the *cytochrome oxidase* subunit II gene (COII) and an 835 bp fragment of the *cytochrome b* gene (*cytb*). PCRs were performed using primer pair combinations COIIF [5’-AATGGCCTAYCCYCTYCAAC-3’], COIIR [5’-CCGCAAATTTCTGARCACTG-3’], and cytbF [5’-CCCACCCCCTCTAACATCTC-3’], cytbR [5’-TAAGGATTGARGCKACAAGG-3’].

PCR amplification was performed in 30 µl reactions containing 1× polymerase reaction buffer, 2 mM MgCl_2_, 0.2 mM dNTPs, 0.03 U *Taq* DNA polymerase (New England Biolabs), 0.5 µM of each primer, and 5 µl of template DNA. Reactions were made up to a final volume of 30 µl with ddH_2_O. PCR amplification of double-stranded product was carried out with an Eppendorf Mastercycler (Eppendorf, Hamburg, Germany). PCR conditions comprised of an initial 7 min denaturing step at 94°C, then 40 cycles of denaturation at 94°C (20 sec), annealing at 54°C (30 sec), and extension at 72°C (60 sec), with a final extension step of 72°C for 1 min preceding an indefinite hold period at 4°C. PCR products were sent to Macrogen laboratories (Seoul, Korea) for purification and sequencing on an ABI3730 XL DNA sequencer (Applied Biosystems, Foster City, CA). To ensure accuracy, all amplified products were sequenced in both the forward and reverse direction using primers from the initial PCR reaction. Of the amplified segments, 518 bp of COII and 738 bp of *cytb* were used in the analysis. Negative controls were used to ensure contamination was avoided.

Individuals sampled from Kangaroo Island in December 2008 and December 2009 were used to assess contemporary levels of genetic diversity. The contribution of the founding individuals was determined by incorporating all Kangaroo Island and Warrawong individuals (sampled between 1991 and 2009). Due to the long-term isolation and low genetic diversity present on King Island, analyses of this population were conducted incorporating all samples (i.e., sampled between 2000 and 2009).

Given the genetic similarity among individuals of the upper Yarra catchment (data not shown), all individuals sampled from this region were analyzed to estimate the genetic characteristics of Kangaroo Island's Victorian founder population. Genetic analysis also revealed similarity between individuals of Tasmania's northwestern catchments (data not shown) and, consequently, all individuals sampled from this region were used to estimate the genetic characteristics of Kangaroo Island's Tasmanian founder population.

For the microsatellite data, fstat version 2.9.3 (Goudet 2001) was used to calculate allelic richness averaged over loci and Weir and Cockerham's (1984) measure of *F*_IS_. Significant differences in allelic richness were calculated by a Wilcoxon signed-rank test (Wilcoxon 1945). Observed (*H*_O_) and expected heterozygosities (*H*_E_) were estimated and deviations from Hardy–Weinberg equilibrium (HWE) were determined by exact tests and permutation in arlequin version 3.11 (Excoffier et al. 2005). A global estimate and population pairwise estimates of *F*_ST_ were calculated in fstat (1500 permutations). A factorial correspondence was implemented in genetix version 4.05 (Belkhir et al. 2004) to visualize differences in genetic diversity between populations. The two factors that explained the majority of the variation in multilocus genotypes were plotted.

Structure version 2.3.3 (Pritchard et al. 2000) was run to determine the relationship between individuals. Using only genetic data, Structure identifies the number of distinct clusters, assigns individuals to clusters, and identifies admixed individuals. To determine the number of populations (*K*) within the data set, five independent simulations of each *K* (1–5) were run with 100,000 burn-in iterations and 500,000 data iterations. The most likely value of *K* is the one that maximizes the log-likelihood of obtaining the observed sample of multilocus genotypes (Pritchard et al. 2000). We used the method of Evanno et al. (2005) to estimate the true *K*. Structurama (Huelsenbeck et al. 2011) was also used to infer the number of populations. This program differs slightly from structure in that it allows you to place a prior distribution on *K* and estimate the number of populations directly.

To determine the contribution of founders on Kangaroo Island, a genetic assignment test was performed using the program *GeneClass2* (Piry et al. 2004). Tasmanian and Victorian individuals were partitioned to create two independent reference populations. Kangaroo Island individuals were assigned to a reference population based on the Bayesian methods of Rannala and Mountain (1997). The probability of correctly assigning individuals to their population of origin was calculated from 1,000,000 Monte Carlo simulations with a Type I error of 0.01. High values indicate a good fit to the assignment population, while values less than about 0.05 suggest a poor fit to the population.

Sequenced mitochondrial products were aligned using mega version 4.0 (Tamura et al. 2007) and edited manually. Levels of genetic differentiation between the haplotypes were calculated using pairwise genetic distances under a Kimura-2-parameter model in mega version 4.0 (Tamura et al. 2007). A haplotype network was constructed in tcs 1.21 (Clement et al. 2000) to visualize base pair changes and relationships between haplotypes.

### Population size modeling—Kangaroo island

Intensive platypus mark-release-recapture studies were carried out along Rocky River in Kangaroo Island. The surveyed area consists of relatively high quality habitat (R. Ellis, unpubl. data) and is likely to contain proportionally more platypuses than regions upstream or downstream. Consequently, despite the surveyed area constituting approximately one-third of the platypus’ known distribution on the island, it is estimated to contain approximately half the population (R. Ellis, unpubl. data). Surveys were carried out roughly every month from 1 March 1998 until 19 November 1999 with a total of 92 capture events recorded across 44 unique adult individuals. For analytical purposes, each sampling month was allocated to a capture occasion. Sex, body weight, head-to-tail length, bill width, bill length, and shield measurements were also recorded for each captured individual. These measurements were normalized prior to model fitting. Data for males and females were analyzed separately. For the female data there were no records between March 1998 to July 1998, May 1999 to July 1999, and for October 1999. To avoid data sparseness, observations from August 1998 and September 1998 were pooled. This gave 40 observations of 20 unique female platypuses across 11 capture occasions. For the male data, there were no records for May 1998, November 1998, February 1999, June 1999, and October 1999. We pooled observations for male data for the months March/April for both years 1998 and 1999. This gave 52 observations of 24 unique male platypuses across 14 capture occasions. Individual capture histories were then constructed and covariates were taken as the first recorded observation and assumed constant across each capture occasion.

The population size for platypus within the surveyed area was denoted as *N*. An estimate of the population size was denoted by 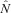 and their standard errors by SE (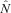), which can be used to obtain 95% confidence intervals (CIs). Each data set was tested for closure because different models are used when the population is assumed closed or open. For closed populations, the population is assumed to remain constant throughout sampling. For open populations, the population is susceptible to births, deaths, immigrations, and emigration and if individuals leave the population, they do so permanently. To test for closure we consider the tests of Stanley and Richards (1999) where we define the Stanley and Burnham (1999) test as Test 1 and the Otis et al. (1978) test as Test 2. In these tests, the null hypothesis assumes closure, so significant (α < 0.05) *P* values suggest the population is not closed.

In closed population models, only the population size and capture probabilities are estimated. Conditional likelihood models were used (Huggins 1989, 1991) where capture probabilities can permit heterogeneity between individuals (*h*), behavioral response (*b*), or time (*t*) (Otis et al. 1978). That is, capture probabilities are modeled linearly as a function of: individual covariates, such as body weight or gender for heterogeneity models, time-effects for time-dependent models, and trap behavior effects for behavioral response models. These models are denoted by *M*_(*)_, for example, model *M*_(tb)_ allows for capture probabilities to depend on time and behavioral response. Also, model *M*_(0)_ assumes all individuals have the same probability of capture. There are eight combinations of these models in total.

For open population models, all individuals are assumed to have the same probability of capture. In addition to estimating the population size, survival and recapture probabilities and new arrivals to the population are also estimated. The classical Jolly–Seber (JS) model (Seber 1982; Pollock et al. 1990) can be used, however, we use the more modern approach of Schwarz and Arnason (1996), which generalizes the way birth is modeled in the usual JS model. This allows an open population estimate to be obtained.

For both models, the estimates are obtained by using capture histories and maximum likelihood estimation (section 1.3 in Amstrup et al. [2005]). The Akaike information criterion (AIC) statistic (Burnham and Anderson 1999) was used for model selection.

The average effective population size over time was estimated from genetic data following the equation of Culver et al. (2008).

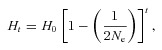

where *H_t_*= expected heterozygosity in generation *t*, *H*_0_= initial heterozygosity, and *N*_e_= the effective population size.

The effective population size on Kangaroo Island was calculated according to the equation *N*_e_/*N*= 0.1 (Frankham 1995b). A simple simulation was undertaken for Kangaroo Island's population to predicted changes in heterozygosity and allelic richness over time. The simulation was undertaken in PopTools (Hood 2002) using random sampling of allele frequency data each generation for a given *N*_e_. The simulation assumes random mating, nonoverlapping generations, and a constant effective population size across time.

## Results

### Genetic diversity

A total of 136 alleles were detected across 13 polymorphic microsatellite loci. All loci were polymorphic in Tasmania while Victoria had one monomorphic locus (*OA* 11.9), Kangaroo Island had two (*OA* 11.9 and *OA* 10.5), and King Island had eight monomorphic loci (Table 1). The majority of all alleles identified (>85%) were present in the Victorian population. There was a clear genetic distinction between individuals of Victoria and Tasmania with unique alleles observed at 12 loci. Victoria's population contained 72 unique alleles and Tasmania's population contained 18 unique alleles (Table 1). No unique alleles were present in the King Island samples; all alleles were present in Victoria's upper Yarra population and incorporated several alleles unique to Victoria. Similarly, nearly all the alleles identified from Kangaroo Island were present in Victoria's upper Yarra individuals and incorporated several alleles unique to Victoria. One allele was detected on Kangaroo Island unique to this population. Neither Kangaroo Island nor King Island contained any of the alleles identified as being unique to Tasmania.

**Table 1:** Thirteen microsatellite loci screened, primer sequences, annealing temperatures, and the number of alleles found in the contemporary populations of upper Yarra Victoria, northwestern Tasmania; Kangaroo Island, South Australia; and King Island, Tasmania. Unique alleles are shown in brackets with alleles unique to northwestern Tasmania in italics and alleles unique to Victoria's upper Yarra population in bold text.

Locus	Repeat motif	Primer sequence 5’-3’	*T*_a_	Upper Yarra Victoria (*n*= 60)	Northwestern Tasmania (*n*= 19)	Kangaroo Island (*n*= 12)	King Island (*n*= 21)
OA 1.3	(GA)_5_ N (AG)_5_ N_20_ (AG)_14_	F: GACCTCTTTGCCACTTTGCTA	59.4	10 (**6**)	5 (*1*)	2 (**1**)	1
		R: GGATTAGAACCCACGATCTGTT					
OA 3.2	(GA)_5_ (AT)_5_ N_10_ (TG)_12_	F: GCCCTATGTACCTTGAATATAA	48.7	6 (**3**)	3	2	2
		R: ACAGTTGGTGGACTTGATTC					
OA 4.5	(AC)_14_	F: ACGCCCCACCCGTTCCCTTTC	61.4	6 (**2**)	7 (*3*)	2	1
		R: ATCCATTCGCCGATCTCCTGTGC					
OA 5.1	(GT)_14_	F: CTTGGAAAGCATACACAGATG	52.0	4 (**2**)	2	3 (**1**) (1)*	1 (1)
		R: GAAATTGTTGGACTATGGGTAT					
OA 6.2	(TC)_22_	F: TAGGGTGGTTTGAAAGGTTTTG	56.1	18 (**14**)	9 (*5*)	5 (**5**)	1 (**1**)
		R: AGACAGCCGTAGGAGCACTAAA					
OA 7.3	(TG)_12_	F: AATCTGAAAAGGCAACAATCT	48.4	12 (**8**)	5 (*1*)	3 (**1**)	2 (**1**)
		R: GGGCTTATCATTTGTCCTCTA					
OA 10.5	(TC)_9_ N (CA)_10_	F: GCTCTGATGGCTAATACTGCTA	50.3	3	3	1	1
		R: ATCCCTTCCCTCTCCATTATTA					
OA 11.9	(GA)_5_(GT)_10_	F: GGTCAAAGAGTCCCAGAATGAC	60.1	1 (**1**)	2 (*2*)	1 (**1**)	1 (**1**)
		R: GAGACAGGAAACTTGGCATAGG					
OA 12.6	(CA)_14_	F: GATCTCCCACTACCGACAGTTT	56.1	9 (**7**)	3 (*1*)	5 (**4**)	3 (**1**)
		R: CAGGGTGGAATGATTACAGAAA					
OA 14.3	(CA)_14_ (CACAC)_2_	F: GAAGGAGGAGGAGAGGTTGACA	54.0	14 (**10**)	5 (*1*)	3 (**2**)	1
	(AC)_5_ (CA)_13_	R: TTCAGCGACTTTTCTGTTCCATAG					
OA 17.6	(GA)_10_	F: GTAACTTCTCACGGGGCAACTT	53.5	8 (**6**)	3 (*1*)	2	2
		R: GGCATTTTATTTTCTCGCCTCTA					
OA 18.5	(TG)_11_ N_3_ (GC)_6_	F: TTGTCTATATTCTTGGAAGGGCTC	60.0	10 (**6**)	5 (*1*)	3 (**1**)	1 (**1**)
		R: ATTGCAGGTAAAGTGAAGGGAA					
OA 20.12	(TC)_15_(CT)_17_	F: GTTCCCTTGAGGACGGAGA	60.1	16 (**7**)	11 (*2*)	5 (**3**)	2
		R: CAGTGGGCTTTTCCATTCATA					

*T*a, annealing temperature; number of alleles observed (number of unique Tasmanian alleles are given in parenthesis).

* Allele not present in either upper Yarra or northwestern Tasmanian individuals sampled—broader sampling has detected this allele in other contemporary mainland populations including <50 km from the upper Yarra population (data not shown).

Genetic diversity estimates were similar in the Tasmanian and Victorian populations; expected heterozygosities were 0.606 in Tasmania and 0.597 in Victoria. Allelic richness estimates differed significantly between Victoria and Tasmania (*P* < 0.01) with values of 4.34 and 5.79, respectively (Table 2). Genetic diversity estimates of the contemporary Kangaroo Island population (i.e., sampled 2008–2009) revealed a lower level of allelic richness (*r*= 2.85) and expected heterozygosity (*H*_E_= 0.419) compared with Tasmanian and Victorian populations. King Island exhibited extremely low genetic diversity, with an allelic richness of 1.32 and expected heterozygosity of 0.032 (Table 2). Inbreeding levels were also high within the King Island population (*F*_IS_= 0.205, *P* < 0.01) but the population was not out of HWE, which is likely due to low sample size and extremely low numbers of alleles. In comparison to Victoria, King Island and Kangaroo Island contained few rare alleles (mean 0.462 and 0.692, respectively). Numbers of rare alleles in Tasmania were also comparatively low (mean 1.154).

**Table 2:** *Ornithorhynchus anatinus* population genetics statistics for individuals sampled from the upper Yarra Victoria, northwestern Tasmania, King Island Tasmania, and South Australia. Samples from South Australia have been divided into categories to calculate genetic diversity in each of two groups: the most recent Kangaroo Island population (sampled from 2008 to 2009) and all Kangaroo Island and Warrawong Sanctuary individuals (sampled from 1991 to 2009). All individuals were genotyped at 13 microsatellite loci.

Region	*n*	*a*	*r*	*H*_O_	*H*_E_	*F*_IS_	HWE*P-*value	r*A*
Upper Yarra, Victoria	60	9.00	5.76	0.549	0.597	0.08	0.646	5.077 (±3.499)
Northwestern Tasmania	19	4.85	4.34	0.550	0.606	0.095	0.606	1.154 (±1.463)
King Island, Tasmania	21	1.46	1.32	0.026	0.032	0.205	0.083	0.462 (±0.660)
Kangaroo Island, South Australia, 2008–2009	12	2.85	2.85	0.423	0.419	−0.01	0.495	0.692 (±0.947)
Kangaroo Island/Warrawong Sanctuary, South Australia	25	3.54	3.02	0.395	0.431	0.086	0.467	1.077 (±1.256)

*n*, the number of individuals genotyped for each population; *a*, mean number of alleles; *r*, allelic richness; *H*_O_, observed heterozygosity; *H*_E_, expected heterozygosity; *F*IS, multilocus estimates; HWE, Hardy–Weinberg equilibrium *P*-values.

* Significance after corrections for multiple comparisons. Locus; r*A*, mean number of rare alleles (frequency ≤ 0.05) per locus.

**Figure 2:**
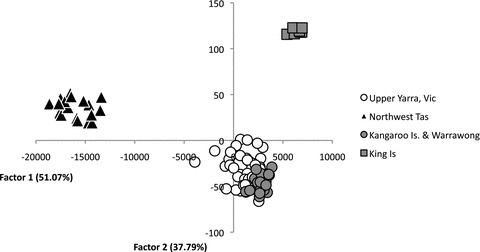
Factorial correspondence analysis depicting the microsatellite genetic difference between *Ornithorhynchus anatinus* individuals of four locations: the upper Yarra, Victoria; northwestern Tasmania; King Island, Tasmania; and Kangaroo Island and Warrawong Sanctuary, South Australia. Differences are represented across two factors with factor 1 representing 51.07% of the variation and factor 2 representing 37.79% of the variation.

Significant genetic differentiation was present between the Tasmanian and Victorian populations with an *F*_ST_ of 0.254 (95% CI, 0.144–0.394). King Island was significantly differentiated from these two populations with an *F*_ST_ of 0.396 with Victoria (95% CI, 0.297–0.478) and 0.635 with Tasmania (95% CI, 0.554–0.715). South Australian platypuses (Kangaroo Island and Warrawong individuals) were only slightly differentiated from Victorian samples (*F*_ST_= 0.079, 95% CI, 0.043–0.114) but highly differentiated from Tasmanian samples (*F*_ST_= 0.366, 95% CI, 0.266–0.476). The factorial correspondence analysis provides a visualization of these differences (Fig. 2), showing the similarity between individuals of Victoria and Kangaroo Island and the clear distinction between individuals of Tasmania, Victoria, and King Island.

**Figure 3:**
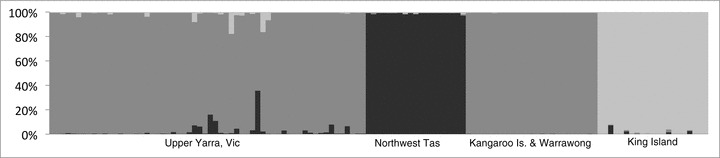
Summary plot of the estimated membership coefficient for each *Ornithorhynchus anatinus* individual across three population clusters (*K*= 3). Each individual is represented by a single vertical line broken into three shaded segments. Segment represents the proportional membership to each of the three population clusters. Individuals are grouped into the regions from which they were sampled: upper Yarra, Victoria; northwestern Tasmania; Kangaroo Island and Warrawong Sanctuary, South Australia; and King Island, Tasmania.

Structure analysis indicated the data set was comprised of three populations (*K*= 3) and this was supported by Structurama. King Island individuals were allocated to a distinct population group, as were Tasmanian individuals. The third population comprised all Kangaroo Island, Warrawong, and Victorian individuals (Fig. 3). Assignment tests assigned all Kangaroo Island individuals to Victoria with a mean probability of 0.86 (range: 0.46–1.00). Tasmania was excluded as the population of origin for all Kangaroo Island individuals.

**Figure 4:**
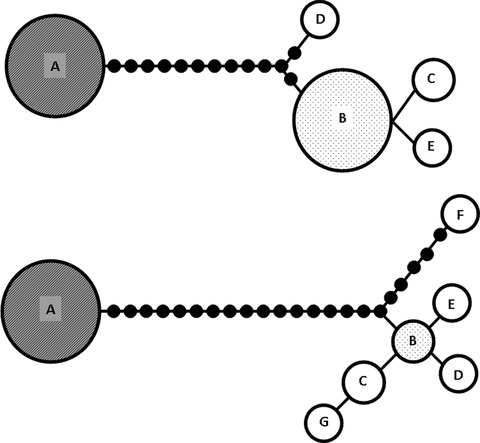
Mitochondrial DNA network analysis showing the relationship between haplotypes for mtDNA gene (a) COII and (b) *cytb*. Dots on branches indicate nucleotide changes. Gray haplotypes indicate presence in Victoria's upper Yarra while white haplotypes indicate presence in northwestern Tasmania. Black stripes identify haplotypes present in Kangaroo Island and Warrawong individuals and black dots indicate haplotypes present in King Island, Tasmania. The number of individuals revealing each COII haplotype are as follows: A = 50; B = 32; C = 3; D = 1; E = 1 and for *cytb*: A = 11; B = 11; C = 4; D = 3; E = 1; F = 1; and G = 1.

mtDNA sequencing produced a clear separation between Victorian and Tasmanian individuals with 11 fixed nucleotide differences at COII and 16 fixed differences at *cytb*. Only one COII and one *cytb* haplotype was present in individuals from Victoria (Fig. 4). The same haplotype was observed in all Kangaroo Island individuals analyzed. Tasmania contained more mtDNA variation with four COII haplotypes and six *cytb* haplotypes (Fig. 4). King Island individuals’ contained only one haplotype at each mitochondrial gene, both of which were present in Tasmania. Kimura-2-parameter distances between COII Victorian haplotype A and Tasmanian haplotypes ranged between 0.0257 and 0.0277, while distances between *cytb* Victorian haplotype A and Tasmanian haplotypes ranged from 0.0236 and 0.0293. Genetic differentiation within Tasmanian samples was low with COII haplotype (B, C, D, E) differences ranging between 0.0019 and 0.0098, while *cytb* haplotype (B, C, D, E, F, G) differences ranged from 0.0014 to 0.0124.

### Population size estimates—Kangaroo island

Each data set (females only and males only) was tested for closure (Table 3). The female data did not indicate lack of closure, thus, we assume the female population is closed. For the male data, the two tests reported different conclusions; therefore, open and closed models were considered.

**Table 3:** Closure tests for the female and male data. Low *P* values indicate lack of closure.

Data	Test 1, *P*-value	Test 2, *P*-value	Conclusion
Male	0.02	0.49	Possibly open
Female	0.08	0.40	Closed

For the female data, all eight closed population models of Huggins (1991) were fitted using the body weight covariate for *M*_(h)_ type models. Other covariates were not used to avoid collinearity and body weight measurements were normalized prior to model fitting. Based on the AIC, model *M*_(0)_ provided the best fit but models *M*_(b)_ and *M*_(h)_ may also be appropriate (Table 4). In order to compare the models fitted to female and male data (see below), a further analysis of model *M*_(h)_ was carried out by considering a quadratic relationship between the covariate and capture probability, however, this did not improve the model fit. Fitted capture probabilities for Model *M*_(h)_were plotted against body weight with CIs. The relationship was very flat and close to a constant model, which may explain why model *M*_(0)_ provided a better fit. According to model *M*_(0)_, the population size of females within the sampled area on Kangaroo Island was estimated as 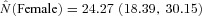 (Table 4).

**Table 4:** Various parametric models fitted to female and male Kangaroo Island platypus data with the body weight covariate for *M*_(h)_-type models. Note that 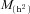 represents a quadratic model.

Data	Model	*M*_(tbh)_	*M*_(bh)_	*M*_(tb)_	*M*_(th)_	*M*_(h)_	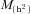	*M*_(b)_	*M*_(t)_	*M*_(0)_
Female	AIC	221.18	204.40	219.55	220.52	203.41	204.75	202.59	218.56	201.46
	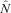	20.75	21.73	20.90	24.21	24.29	24.74	21.83	24.18	24.27
	SE 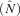	1.42	2.17	1.68	2.92	2.97	3.64	2.28	2.90	2.94
Male	AIC	299.38	286.09	299.55	297.71	285.88	284.74	286.22	297.76	285.87
	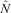	25.98	25.58	25.75	28.15	28.43	32.22	25.27	27.42	27.68
	SE 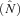	3.05	1.94	2.93	2.95	3.07	6.25	1.63	2.41	2.52

For the male data, both closed and open population models were fitted. Initially, the open population model described above using program MARK (White and Burnham 1999) was fitted. The open population estimate was 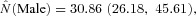 however, identifiability issues were encountered for most model parameters due to sparseness of data. The results for closed population models that also use a normalized body weight covariate are shown in Table 4. The best selected models were *M*_(0)_ and *M*_(h)_. A quadratic model was fitted, which gave a lower AIC and indicated some nonlinearity. If we consider a closed population, the estimated number of male platypuses within the surveyed area was 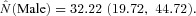 As male platypuses are typically more mobile than females (Grant 2007), it may be more appropriate to consider the male population open. In any case, the estimated population sizes for closed and open population models are similar at approximately 31–32 individuals.

In total, a population of approximately 55 individuals was estimated within the study area. As this study area is assumed to contain approximately half the platypus population of the island, the total population of the island is expected to be roughly double this figure (i.e., ∼110 individuals). This equates to an effective population size of around 11 individuals (*N*_e_/*N*= 0.1).

To estimate the effective population size from genetic data, we use the formulae of Culver et al. (2008). The genetic diversity present in today's Kangaroo Island population is 0.419 (*H_t_*= 0.419). We expect the initial level of heterozygosity in the Kangaroo Island population to be similar to that present in the founding population in the upper Yarra River today (*H*_0_= 0.597). Given platypuses are capable of breeding in their second year and have been recorded surviving up to 21 years of age (Grant 2004), the generation time is assumed to be ∼10 years (although this is likely to contain some degree of error). Approximately seven generations (*t*= 7) have passed since Victorian platypuses were first introduced to Kangaroo Island. This equates to an effective population size of 10.14 or ∼10 individuals, a value similar to that produced by population size models.

**Figure 5:**
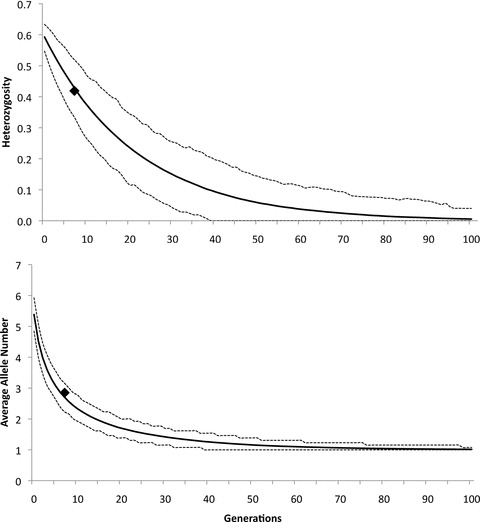
Simulated loss of genetic diversity over time (in generations) of *Ornithorhynchus anatinus* individuals on Kangaroo Island, South Australia (*H*_E_, average heterozygosity and *A*, average allele number). The population was assumed to have commenced with levels of genetic diversity similar to that found in the present day upper Yarra population in Victoria. One thousand Monte Carlo simulations were run for 13 microsatellite loci assuming random mating, nonoverlapping generations and *N*_e_= 11. Solid lines indicate means and dotted lines represent 95% confidence intervals. The current genetic diversity of the Kangaroo Island population is indicated by a black diamond.

Using a simple simulation with effective population size (*N*_e_) of 11, genetic diversity is predicted to continue to decline within Kangaroo Island's platypus population through time (Fig. 5). Within 50 generations, expected heterozygosity and average allele numbers are anticipated to be similar to those observed in King Island's platypus population.

## Discussion

Levels of genetic diversity in platypus populations from Tasmania and Victoria are similar to those observed in mainland populations of other Australian mammalian species (Taylor et al. 1994; Pope et al. 2000; Bowyer et al. 2002; Eldridge et al. 2004). The sampled region of Tasmania does, however, possess a reduction in allelic richness and lower frequency of rare alleles in comparison to Victoria's upper Yarra population but mitochondrial DNA diversity remains high. On Kangaroo Island, levels of genetic diversity are reduced with ∼30% decrease in expected heterozygosity and ∼50% decrease in allelic richness in comparison to Victoria. The genetic diversity of King Island is extremely low with a high proportion of monomorphic loci, ∼70% reduction in allelic richness, and >90% reduction in expected heterozygosity in comparison to Victoria. These microsatellite genetic diversity estimates for King Island platypuses are among the lowest ever recorded for a naturally outbreeding vertebrate population.

Island populations frequently have reduced genetic diversity in comparison to mainland populations (Frankham 1997, 1998). The rate of loss of genetic diversity is dependent on the effective population size (*N*_e_) and the number of generations over which the population has been isolated (Frankham 1997). Consequently, reductions in genetic diversity are often more pronounced in endemic rather than relatively recently introduced or translocated populations (e.g., Cardoso et al. [2009]). King Island has been isolated from mainland Australia for close to 12,000 years and isolated from Tasmanian for ∼10,000 years (Blom 1988). This long-term isolation combined with small population size has likely contributed to the severe reduction in genetic diversity found in this *O. anatinus* population. While no gross morphological changes have been detected (i.e., length, weight, external appearance), additional phenotypic traits have not been assessed within this population and therefore it is not known if the severely low levels of genetic diversity are impacting population fitness (inbreeding depression). In any case, the low levels of genetic diversity suggest that this population is exposed to an increased risk of extinction (Markert et al. 2010), and if it is challenged by environmental stress and/or parasites and disease, platypuses may become extinct on King Island.

Determining the relationship between King Island's platypuses with those of Tasmania or Victoria is difficult because contact with the major land masses to the north and south was lost ∼10,000 years ago. Although King Island retains a mtDNA haplotype present in Tasmania, it also contains several nuclear alleles that are unique to today's Victorian population. Thousands of years of independent evolution will have seen allele frequencies and distributions alter significantly on both island and mainland populations. For the small King Island population, this extended period of isolation has also lead to extremely low genetic diversity, low allele number, and consequently, its identification as a distinct population.

The greater proportion of heterozygosity retained on Kangaroo Island (∼70%) is likely to reflect this population's relatively recent isolation, around 70 years ago. The present-day population on Kangaroo Island, however, is likely to be limited in its population size. From a small number of founders, the Kangaroo Island population has undergone population growth, attaining an estimated size of ∼110 individuals by 1998/1999. An extremely low proportion of juveniles encountered during trapping periods (<5% in comparison to nearly 33% on the mainland [Grant 2004]) appears to indicate that the population may have reached carrying capacity within its current distribution along the Rocky and Breakneck Rivers. Despite records of platypuses traversing overland on the island (Ellis 2000), individuals have yet to be encountered in waterways outside of the two they were initially introduced into. There may be potential for the population to expand their range into other waterways on the island and thus increase the total population size. Within their current distribution, however, the population is unlikely to grow beyond 110 individuals. The small number of founders, genetic isolation, and sustained small population size will continue to impact on the genetic diversity of this island population. Both microsatellite and mitochondrial data only provide support for the contribution of Victorian founders suggesting the initial Tasmanian founders were unsuccessful. This limits the founder population to a maximum of 16 Victorian individuals and potentially fewer effective founders (those that have contributed genetically to subsequent generations).

Today's Kangaroo Island platypus population remains small 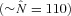 and the number of individuals contributing to subsequent generations is likely to be even smaller. Estimates of the effective population size of Kangaroo Island obtained from both population size modeling and genetic data were similar, indicating an *N*_e_ of approximately 10–11 individuals. This is extremely low and potentially unsuitable for long-term population persistence. An *N*_e_ of at least 500 (Franklin 1980), but more likely greater than 1000 (Willi et al. 2006; but see [Lande 1995]) has been proposed as a minimum to maintain the evolutionary potential of a population. At *N*_e_ < 50, inbreeding is likely to be high and can limit a population's ability to adapt even in the short-term (Franklin 1980). Recently bottlenecked populations are likely to experience a reduction in allele number, particularly due to the loss of rare alleles, but can still maintain reasonable levels of heterozygosity in the short-term (Luikart et al. 1998). This is in line with the findings on Kangaroo Island where allelic richness (*r*= 2.85) and the proportion of rare alleles are low (r*A*= 0.692) compared to Victoria, whereas heterozygosity levels are reduced but not to the same extent (*H*_E_= 0.419). Isolation since 1946 in combination with a small effective population size is likely to have elevated the impact of genetic drift, contributing to the observed decrease in allele number (particularly the loss of rare alleles) and decrease in heterozygosity.

With a small population size and continued isolation of the Kangaroo Island population, genetic diversity is likely to continue to diminish through time (Fig. 5). Without intervention, it is likely that genetic diversity will continue to decline and be in a similar state to that observed on King Island within 50 generations (∼200–500 years according to generation times based on the average age of breeding females and longevity estimates in Grant [2004]).

Long-term isolation and low *N*_e_ are also likely to have led to inbreeding on these island populations. Islands typically exhibit increased levels of inbreeding with the effective inbreeding (*F*_e_) estimated from equation 1 from (Frankham 1998),

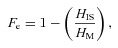
1
where *H*_IS_ is the heterozygosity of the island population and *H*_M_ is the heterozygosity of the mainland population. For *O. anatinus* on Kangaroo Island, the effective inbreeding coefficient is large (*F*_e_= 0.30) and for the King Island population, inbreeding is severe (*F*_e_= 0.95).

Low levels of genetic diversity and high levels of inbreeding are likely to have important consequences for the long-term survival of island populations. Both measures correlate with population persistence as well as various fitness indicators including reproductive success, survival, and parasite resistance (Ralls et al. 1988; Westemeier et al. 1998; Coltman et al. 1999; Crnokrak and Roff 1999; Seymour et al. 2001; Brook et al. 2002; Reed and Frankham 2003). Despite the occurrence of long-term inbreeding on King Island, the genetic load is still likely to be high (Frankham 1995a; Ballou 1997; Eldridge et al. 1999). Indeed, King Island individuals harbor low diversity in an important immune response gene family, the major histocompatability complex (MHC) (Lillie 2009). The occurrence of the fungal mucormycosis on the Tasmanian mainland (Gust and Griffiths 2009) is of particular concern and could have potentially devastating consequences should it emerge within the island population. High levels of genetic diversity are required to maintain adaptive potential and minimise the risk of extinction (Reed and Frankham 2003).

## Conclusion

Islands can act as an important reservoir for Australian wildlife. They are often free from introduced predators or competitors. There are numerous examples of species persisting on islands despite extinction on the mainland (Van Dyck and Strahan 2008). Island populations can provide a valuable source of individuals for conservation programs, including captive breeding, reintroductions, or translocations. However, island populations also present many problems that must be effectively managed if they are to be employed in conservation efforts.

Currently, genetic diversity in King Island *O. anatinus* is severely depauperate and the population is likely to be suffering from reduced fitness, reduced evolutionary potential, and an increased risk of extinction. Consequently, their long-term viability is likely to be under threat. Levels of genetic diversity on Kangaroo Island are reduced in comparison to mainland populations and are predicted to decrease to levels as low as King Island within approximately 50 generations (Fig. 5). To maintain adaptive potential and minimise the risk of extinction (Reed and Frankham 2003), levels of genetic diversity need to be maintained (in the case of Kangaroo Island) or ideally, increased. If additional suitable platypus habitat can be found on Kangaroo Island, increasing the total population size through population range expansion can slow the loss of genetic diversity. Human-mediated migration provides an alternative means to mitigate the negative effects of lowered genetic diversity with the transfer of individuals from suitable source populations. The contribution of just one effective migrant per generation is presumed sufficient to alleviate the effects of drift and reduce inbreeding depression while maintaining local adaptation (Wang 2004). The long-term persistence of these populations will ultimately depend on adequate levels of genetic diversity (Bouzat 2010) and the addition of new genetic material provides a means to achieve that goal.
